# Functional Characterization of the Mannitol Promoter of *Pseudomonas fluorescens* DSM 50106 and Its Application for a Mannitol-Inducible Expression System for *Pseudomonas putida* KT2440

**DOI:** 10.1371/journal.pone.0133248

**Published:** 2015-07-24

**Authors:** Jana Hoffmann, Josef Altenbuchner

**Affiliations:** Institut für Industrielle Genetik, Universität Stuttgart, Allmandring 31, 70569, Stuttgart, Germany; University Paris South, FRANCE

## Abstract

A new pBBR1MCS-2-derived vector containing the *Pseudomonas fluorescens* DSM10506 mannitol promoter P*_mtlE_* and *mtlR* encoding its AraC/XylS type transcriptional activator was constructed and optimized for low basal expression. Mannitol, arabitol, and glucitol-inducible gene expression was demonstrated with *Pseudomonas putida* and *eGFP* as reporter gene. The new vector was applied for functional characterization of P*_mtlE_*. Identification of the DNA binding site of MtlR was achieved by *in vivo* eGFP measurement with P*_mtlE_* wild type and mutants thereof. Moreover, purified MtlR was applied for detailed *in vitro* investigations using electrophoretic mobility shift assays and DNaseI footprinting experiments. The obtained data suggest that MtlR binds to P*_mtlE_* as a dimer. The proposed DNA binding site of MtlR is AGTGC-N_5_-AGTAT-N_7_-AGTGC-N_5_-AGGAT. The transcription activation mechanism includes two binding sites with different binding affinities, a strong upstream binding site and a weaker downstream binding site. The presence of the weak downstream binding site was shown to be necessary to sustain mannitol-inducibility of P*_mtlE_*. Two possible functions of mannitol are discussed; the effector might stabilize binding of the second monomer to the downstream half site or promote transcription activation by inducing a conformational change of the regulator that influences the contact to the RNA polymerase.

## Introduction

Pseudomonads thrive in various ecological niches such as soil, plants, rhizosphere, water bodies, humans, and animals. They possess the metabolic power to utilize a remarkably broad range of substrates, including carbohydrates, fatty acids, organic acids, alcohols, amines, amino acids as well as aromatic and aliphatic hydrocarbons [1].

**Fig 1:**
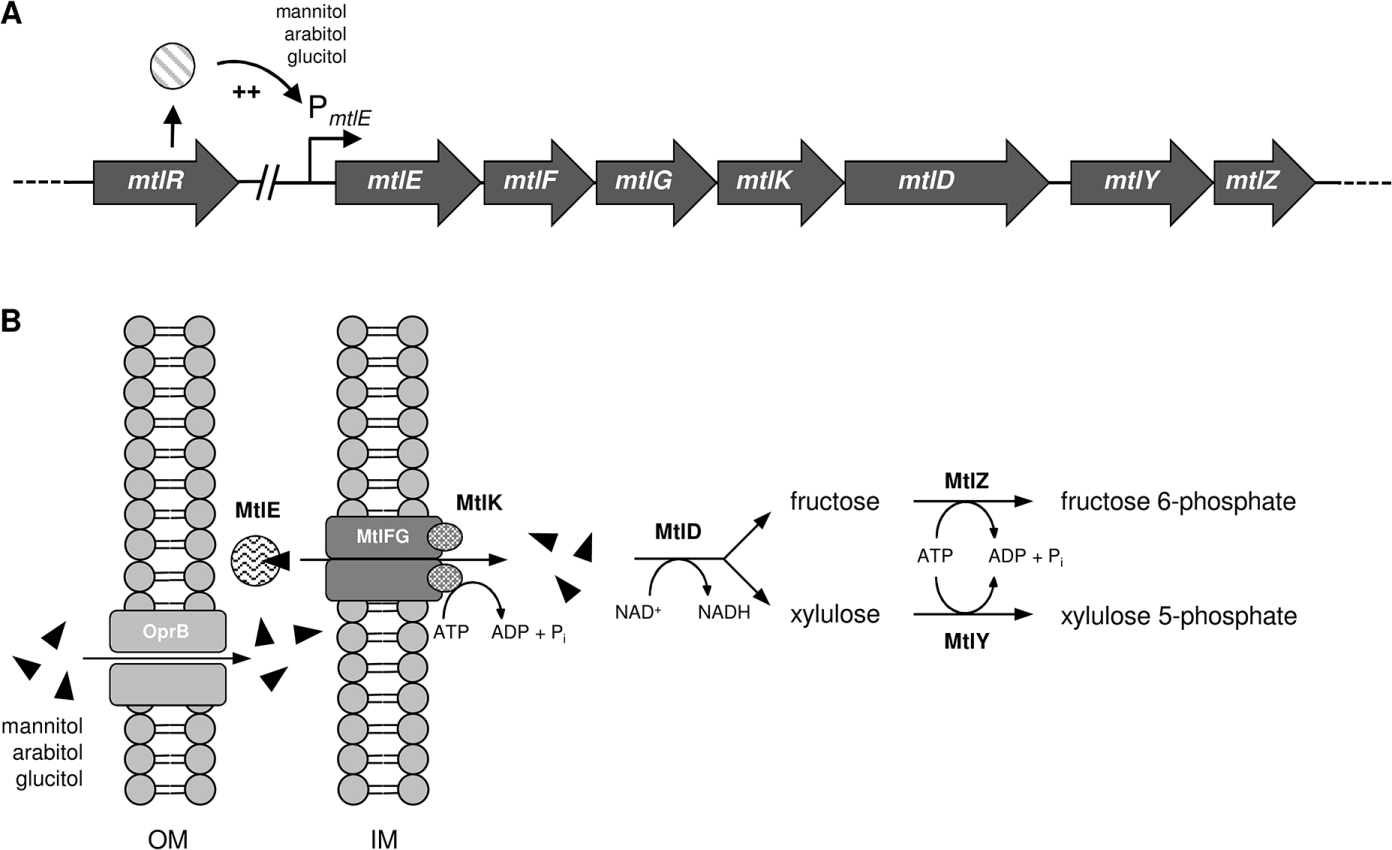
Mannitol, arabitol and glucitol utilization by *Pseudomonas fluorescens* DSM10506. (A) Structure of the mannitol operon. In the presence of mannitol, arabitol, or glucitol, transcription of *mtlE-Z* is activated by MtlR. *mtlR* is located apart from the other genes in the genome of *P*. *fluorescens* DSM10506. (B) Mannitol, arabitol and glucitol are translocated into the periplasm likely by outer membrane porin OprB. MtlE (periplasmatic binding protein), MtlFG (transmembrane domains) and MtlK (ATP binding cassette domain) mediate specific transport of the polyols into the cytoplasm where they are oxidized by MtlD (mannitol 1-dehydrogenase). The products fructose (produced from mannitol and glucitol) and xylulose (produced from arabitol) are phosphorylated by MtlZ (fructose kinase) and MtlY (xylulose kinase) and thus trapped inside the cell. OM = outer membrane, IM = inner membrane.

Mannitol is naturally produced by numerous creatures including plants, fungi, brown algae, yeasts, and bacteria and as a result is the most abundant sugar alcohol in nature. In contrast to *Pseudomonas putida*, *Pseudomonas fluorescens* strains can grow with mannitol as sole carbon and energy source. The mannitol utilization genes of *P*. *fluorescens* DSM50106 are organized in an operon consisting of seven catabolic genes encoding the components for mannitol transport and conversion [2,3]. The operon components also mediate transport and utilization of glucitol and arabitol (Fig 1). The three polyols enter the periplasm likely *via* OprB, an outer membrane porin for monosaccharide uptake in pseudomonads [4,5]. Translocation of mannitol, arabitol, and glucitol into the cytoplasm is mediated by an ABC transporter encoded by *mtlE*, *mtlF*, *mtlG*, and *mtlK*. The mannitol dehydrogenase MtlD oxidizes the three polyols to the corresponding keto sugars. Fructose and xylulose are phosphorylated by kinases MtlY and MtlZ and thus channeled into the intermediary metabolism.

Transcription of the *mtlE*-*Z* operon is positively regulated in an effector-dependent manner [3]. In the presence of mannitol, arabitol, or glucitol, transcription of *mtlE*-*Z* is stimulated by MtlR. Located apart from *mtlE*-*Z*, *mtlR* encodes a protein composed of 301 amino acid residues that comprises sequence similarity to the AraC/XylS family. Transcriptional regulators of the AraC/XylS type are widely distributed among Gram-negative and Gram-positive bacteria modulating transcription of genes with diverse biological functions such as metabolism, stress response, virulence, or bacteria-plant interaction [6–10]. They typically comprise 250–300 amino acid residues and share a conserved DNA binding domain of about 100 residues that is usually located at the C-terminus. The majority of AraC/XylS regulators additionally possess a non-conserved N-terminal domain responsible for effector recognition and dimerization [6,11]. AraC/XylS type regulators involved in stress response are known to be active as monomers (for example SoxS, Rob) [7] while those associated with carbon metabolism often form dimers (for example AraC, XylS, MelR) [12–15].

The AraC/XylS type DNA binding domain is composed of two helix turn helix (HTH) motifs connected by a central helix. The recognition helices of each HTH motif are inserted into the major grooves of two adjacent turns of the DNA upon binding [16]. The DNA sequences that are contacted by the recognition helices have been identified for several AraC/XylS family regulators. Rob and related transcription factors like SoxS and MarA were shown to bind asymmetric DNA sequences of about 15 bp length located within the promoter regions of the regulated genes. Rob binds to the motif GCAC-N_7_-CAA [17] and the SoxS binding site is GCAC-N_7_-TAAA [18]. In case of dimeric AraC/XylS regulators, consecutive sites are present each bound by the HTH motifs of one monomer. The XylS binding site is TGCA-N_6_-GGNTA-N_6_-TGCA-N_6_-GGNTA [19,20]. The MekR binding site was identified as CACC-N_5_-CTTCAA-N_6_-CACC-N_5_-CTTCAA [21]. When arabinose is present, the AraC dimer binds to the half sites TAGC-N_7_-TCCATA and TAGC-N_7_-ACCTGA [22,23].

Effector binding causes structural changes of the regulators enabling them to modulate transcription by means of altered DNA binding affinity, DNA bending, and interactions with RNA polymerase [11,24]. For example, both RhaR and RhaS undergo structural changes when they bind their common effector rhamnose but the allosteric mechanisms underlying transcription activation seem to be different. Experimental and modelling data obtained with RhaR and RhaS mutants suggest that binding of rhamnose increases the DNA binding affinity of RhaS, while in case of RhaR an improved contact to the RNA polymerase causes transcription activation [25].

For gene expression in *P*. *putida* KT2440, there exist a number of vectors based on replicons pBBR1, RK2, RSF1010, pUCP, or pNI10 [26–30]. The available promoters for regulated gene expression include LacI^q^/P_*trc*_, XylS/P_m_, RhaR-RhaS/P_*rhaBAD*_, P_T7_, and MekR/P_*mekA*_. [21,31–35]. Although the systems are well-established and definitely valuable tools for gene expression studies in *P*. *putida*, they comprise significant disadvantages like high basal expression (LacI^q^/P_*trc*_) or requirement of applying toxic, volatile (*m*-toluic acid, methyl ethyl ketone), or very expensive (L-rhamnose) inducers. Here, we report the construction of a new expression system for *P*. *putida* based on pBBR1MCS-2 and MtlR/P_*mtlE*_ regulator/promoter system from *P*. *fluorescens* DSM10501. The new system stands out due to its low basal expression and the possibility to use mannitol as cheap and non-toxic inducer. Furthermore, this study investigated some issues that were left unresolved by the work of Brünker *et al*. [2,3]. In detail, the transcription start site of P_*mtlE*_ was determined and the MtlR binding site was investigated by mutational analyses, electrophoretic mobility shift assay (EMSA) and DNase I footprinting.

## Materials and Methods

### Materials and standard procedures

Pure chemicals were purchased from Sigma-Aldrich Labor Chemie GmbH (Steinheim, Germany) or VWR International GmbH (Darmstadt, Germany). DNA digestion, ligation, transformation, and protein analysis were performed according to Sambrook *et al*. [36]. Electroporation of *P*. *putida* was performed as described by Dennis and Sokol [37]. PCRs and cDNA were purified with the NucleoSpin Gel and PCR Clean-up Kit (Macherey-Nagel, Düren, Germany). Plasmids were isolated with the innuPREP Plasmid Mini Kit (Analytik Jena, Jena, Germany). European agar, tryptone, and yeast extract were purchased from BD (Heidelberg, Germany). Restriction enzymes and DNase I were purchased from New England Biolabs (Frankfurt am Main, Germany). T4 DNA ligase was purchased from Roche (Grenzach-Wyhlen, Germany). Oligonucleotides were synthesized by Eurofins MWG (Ebersberg, Germany). All oligonucleotides used in this study are listed in S1 Table. DNA sequencing was performed by GATC Biotech (Konstanz, Germany).

### Bacterial strains, media and culture conditions

*Escherichia coli* JM109 [38] and *Escherichia coli* RR1 [39] *dam* ^-^ were used for plasmid propagation and preparation. *Escherichia coli* HB101 [40] was used for heterologous *mtlR* expression and fluorescence measurement of P_*mtlE*_-*eGFP* fusions. *Pseudomonas putida* GN146 (Δ*upp* (*pp_0746*), Δ*lapABC* (*pp_0166–0168*), Δ*cheW*-*flgN* (*pp_4333*-*pp_4396*), *attB*) was used for fluorescence measurement of P_*mtlE*_-*eGFP* fusions. *P*. *putida* GN146 is a deletion mutant of *P*. *putida* KT2440 [41] lacking biofilm and flagellar biosynthesis genes. The deletions were created with the *upp* deletion system [42].

*E*. *coli* and *P*. *putida* strains were cultivated in Luria-Bertani (LB) medium (10 g l^-1^ tryptone, 5 g l^-1^ yeast extract, 5 g l^-1^ NaCl) in Erlenmeyer flasks at 30°C (*P*. *putida*) or 37°C (*E*. *coli*) and 200 rpm on a rotary shaker. Cultures were supplemented with 50 μg ml^-1^ kanamycin or 100 μg ml^-1^ ampicillin as appropriate.

### Plasmid construction

All plasmids used or constructed in this study are summarised in Table 1. The oligonucleotides used for plasmid construction can be found in S1 Table. For the construction of pJH175.1 (*mtlR* with 5’-*Strep*-tag II fusion), pJH176.2 (*mtlR* wild type), and pJH204.1 (*mtlR* with 3’-*Strep*-tag II fusion), the *mtlR* gene from *P*. *fluorescens* DSM 50106 was amplified by PCR in a total reaction volume of 50 μl containing 250 ng chromosomal DNA, 1 U Phusion Hot Start DNA polymerase (Thermo Scientific, Dreieich, Germany), 1 μM oligonucleotides, 3% (v/v) dimethyl sulfoxide (DMSO), 2 mM dNTPs, and 10 μl 5× GC reaction buffer for Phusion Hot Start DNA polymerase. The PCR products were purified, digested with *Bam*HI/*Hin*dIII (pJH175.1), *Nde*I/*Hin*dIII (pJH176.2), or *Bam*HI/*Nde*I (pJH204.1) and ligated with the 3,548 bp *Bam*HI/*Hin*dIII fragment of pJOE6090.1 (pJH175.1), 3,507 bp *Nde*I/*Hin*dIII fragment of pJOE5751.1 (pJH176.2), or 3,554 bp *Bam*HI/*Nde*I fragment of pJOE6089.4 (pJH204.1), respectively.

**Table 1:** Plasmids used in this study.

Plasmid	Oligonucleotides	Vector used for construction, properties	Reference
**pBTac1**		pBR322, Ap^R^, Tc^R^, *rop*, P_*tac*_, *rrnB*	[43]
**pETR260.1**		RSF1010, Km^R^, *repABC*, P_*mtlE*_, *luc*	[2]
**pETR267**		lambda RESIII phage vector, *mtlR*	[3]
**pJeM1**		pBBR1MCS-2, Km^R^, *mob*, *rep*, *rhaR*, *rhaS*, P_*rhaBAD*_, 6-his-*eGFP*, *rrnB*	[32]
**pJOE4776.1**		pBBR1MCS-2, Km^R^, ΔP_*lac*_, Δ*lacZ*α	[32]
**pJOE4786.1**		pUC18, Ap^R^, *lacPOZ‘*	[32]
**pJOE5751.1**		pBR322, Ap^R^, *rop*, P_*rhaBAD*_, *eGFP*, *rrnB*	[44]
**pJOE6089.4**		pBR322, Ap^R^, *rop*, P_*rhaBAD*_, *eGFP*-*Strep*-tag II, *rrnB*	[44]
**pJOE6090.1**		pBR322, Ap^R^, *rop*, P_*rhaBAD*_, *Strep*-tag II-*eGFP*, *rrnB*	this study
**pJOE7771.1**		pBBR1MCS-2, Km^R^, *mob*, *rep mtlR*, P_*mtlE*_, 6-his-*eGFP*, *rrnB*	this study
**pJOE7784.1**		pBBR1MCS-2, Km^R^, *mob*, *rep*, *rhaR*, *rhaS*, P_*rhaBAD*_, 6-his-*eGFP*, *rrnB*	this study
**pJOE7801.1**		pBBR1MCS-2, Km^R^, *mob*, *rep*, *tetR*, P_*tetA*_, 6-his-*eGFP*, *rrnB*	this study
**pJH165.1**	S8399/S8400	pJOE4786.1, for transcription start site determination	this study
**pJH165.8**	S8399/S8400	pJOE4786.1, for transcription start site determination	this study
**pJH165.13**	S8399/S8400	pJOE4786.1, for transcription start site determination	this study
**pJH165.18**	S8399/S8400	pJOE4786.1, for transcription start site determination	this study
**pJH175.1**	S8464/S8449	pJOE6090.1, *Strep*-tag II-*mtlR*	this study
**pJH176.2**	S8471/S8449	pJOE5751.1, *mtlR*	this study
**pJH189.1**	-	pJOE7771.1, Δ*mtlR*	this study
**pJH204.1**	S8779/S8471	pJOE6089.4, *mtlR*-*Strep*-tag II	this study
**pJH210.1^*a*^**	S8486/S8534	pJOE7771.1	this study
**pJH215.1^*a*^**	S8534/S8923	pJOE7771.1	this study
**pJH216.1^*a*^**	S8534/S8924	pJOE7771.1	this study
**pJH217.1^*a*^**	S8534/S8925	pJOE7771.1	this study
**pJH218.1^*a*^**	S8534/S8926	pJOE7771.1	this study
**pJH219.1^*a*^**	S8534/S8984	pJOE7771.1	this study
**pJH220.1^*a*^**	S8534/S8985	pJOE7771.1	this study
**pJH221.1^*a*^**	S8534/S8986	pJOE7771.1	this study
**pJH222.1^*a*^**	S8534/S8987	pJOE7771.1	this study
**pJH223.1^*a*^**	S8534/S8988	pJOE7771.1	this study
**pJH224.1^*a*^**	S8534/S8989	pJOE7771.1	this study
**pJH225.1^*a*^**	S8534/S9013	pJOE7771.1	this study
**pJH226.1^*a*^**	S8534/S9014	pJOE7771.1	this study
**pJH227.1^*a*^**	S8534/S9015	pJOE7771.1	this study
**pJH228.1^*a*^**	S8534/S9016	pJOE7771.1	this study
**pJH229.1^*a*^**	S8534/S9017	pJOE7771.1	this study
**pJH230.1^*a*^**	S8534/S9303	pJOE7771.1	this study
**pJH233.1^*a*^**	S8534/S9592	pJOE7771.1	this study
**pJH234.1^*a*^**	S8534/S9593	pJOE7771.1	this study
**pJH253.7^*a*^**	S8534/S10177	pJOE7771.1	this study
**pJH255.1^*a*^**	S8534/S10195	pJOE7771.1	this study
**pJH256.1^*b*^**	S8534/S10215	pJOE7771.1	this study
**pJH257.2^*b*^**	S8534/S10216	pJOE7771.1	this study
**pJH258.1^*b*^**	S8534/S10217	pJOE7771.1	this study

^*a*^ Plasmids with mutations upstream of the -35 sequence of P_*mtlE*_.

^*b*^ Plasmids with mutations in the -35 sequence of P_*mtlE*_.

**Fig 2:**
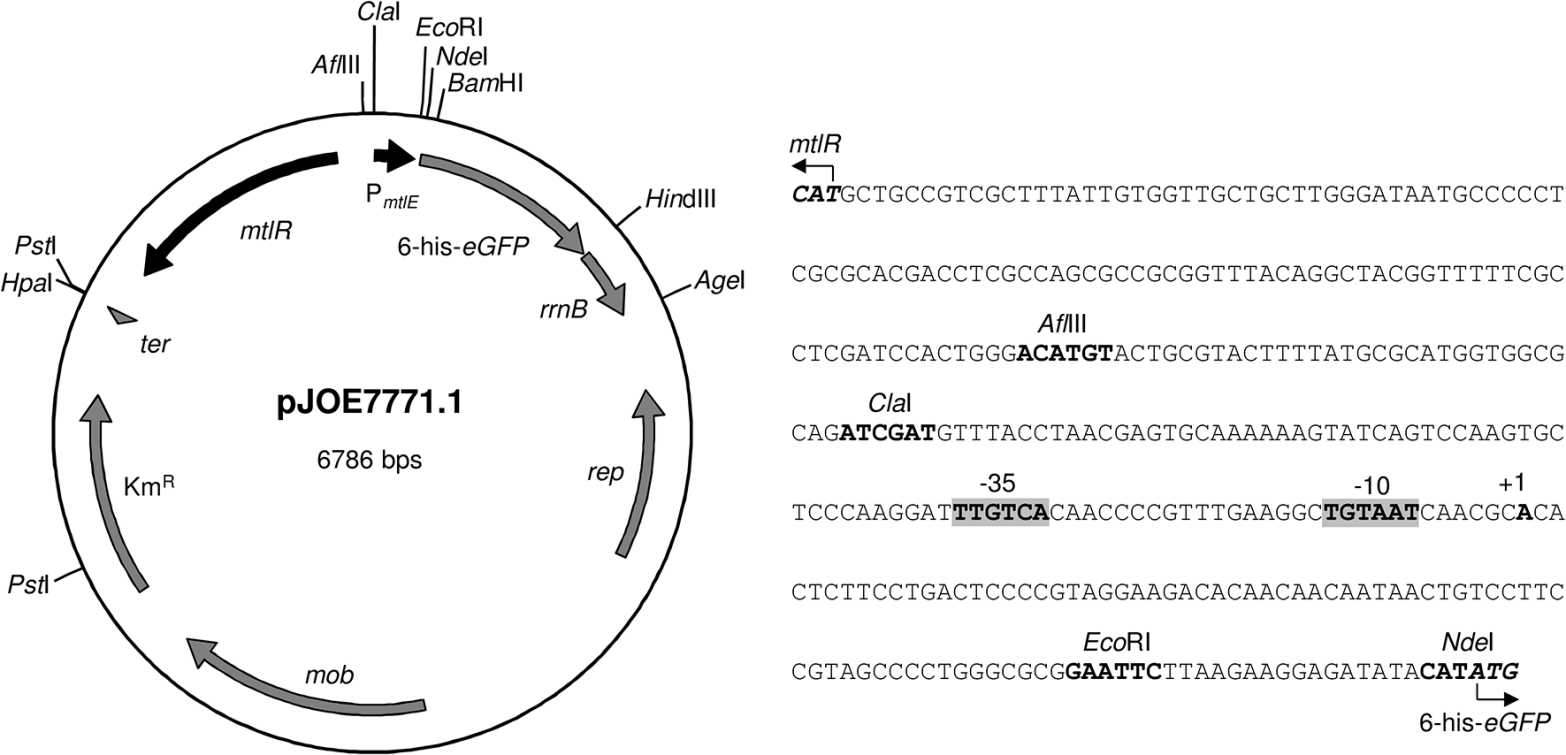
Physical map of pJOE7771.1, a pBBR1MCS-2 derivative with low copy number, and nucleotide sequence of the *mtlR*/6-his-*eGFP* intergenic region. Restriction sites used for the construction of pJOE7771.1, pJH189.1 and the P_*mtlE*_ mutant plasmids pJH210.1-pJH258.1 are shown. Mannitol, arabitol, or glucitol-inducible expression of the reporter gene *eGFP* is mediated by P_*mtlE*_ and *mtlR*. The -10 and -35 boxes of P_*mtlE*_ are indicated in the nucleotide sequence. The transcription start site of P_*mtlE*_ was determined by a modified 5’-RACE protocol (see materials and methods).

The construction of pJOE7771.1 (Fig 2) was performed as follows. In a first step two complementary oligonucleotides (S3510 and S3511) were inserted between the *Eco*RI and *Bam*HI sites of plasmid pBTac1 [43] yielding plasmid pJOE2553.1. The oligonucleotides S3510 and S3511 contain the T7 gene 10 ribosomal binding site, an *Nde*I cleavage site, the first five *lacZ* codons, and 6 histidine codons (CAT). An *eGFP* gene obtained from plasmid pJeM1 by *Bam*HI and *Hin*dIII digestion was fused to the *lacZ*-6 histidine codon sequence by inserting the fragment between the *Bam*HI and *Hin*dIII sites of pJOE2553.1 (resulting in pJOE2713.1). The P_*mtlE*_ promoter sequence was obtained from plasmid pETR260.1 by PCR using the oligonucleotides S3525 and S3526. The PCR fragment was inserted between the *Eco*RI and *Cla*I site of pJOE2713.1 (resulting in pJOE2659.1). The *mtlR* gene was amplified by PCR with the oligonucleotides S3527 and S3528 and pETR267 as template. The amplified fragment was cut with *Cla*I and inserted into pJOE2659.1 (resulting in pJOE2731.1). Next, the *mtlR* gene, P_*mtlE*_, *eGFP*, and the *rrnB* terminator were amplified by PCR with the oligonucleotides S8325 and S8326. The PCR fragment was cut with *Age*I and *Pst*I and inserted into the pBBR1MCS-2 derived vector pJOE4776.1. Finally, the complementary oligonucleotides S3859 and S3869 containing the *rpoS* terminator (*ter*) from *P*. *putida* KT2440 were inserted into the *Hpa*I site to create pJOE7771.1.

In plasmid pJOE7784.1 the *mtlR*/P_*mtlE*_ sequence was exchanced for the *rhaR*-*rhaS*/P_*rhaBAD*_ sequence from pJeM1 using the restriction enzymes *Bam*HI and *Sph*I. Similarly, the *mtlR*/P_*mtlE*_ sequence of pJOE7801.1 was replaced by the *tetR*/P_*tetA*_ sequence from Tn*1721* [45] using *Pst*I and *Bam*HI.

pJH189.1 was created by *Hpa*I/*Afl*III digestion of pJOE7771.1, isolation of the 5,609 bp fragment, Klenow fill-in reaction, and subsequent religation. For the construction of the P_*mtlE*_ mutant plasmids, several PCRs were performed, purified, digested with *Cla*I/*Eco*RI, and ligated with the 6,629 bp *Cla*I/*Eco*RI fragment of pJOE7771.1. For the construction of pJH210.1, the PCR fragment was digested only with *Eco*RI and integrated into pJOE7771.1 (*Cla*I-Klenow filled-in/*Eco*RI). The PCRs were performed as described above with 0.4 ng pJOE7771.1 as template and the oligonucleotides listed in Table 1 and S1 Table. All plasmids were checked by DNA sequencing.

### Fluorescence measurement of eGFP

5 ml LB were inoculated with a single colony of *P*. *putida* GN146 or *E*. *coli* HB101 strains and incubated overnight at 30°C or 37°C, respectively. Next, 15 ml LB were inoculated with the overnight cultures starting from an initial optical density at 600 nm (OD_600_) of 0.07 (*P*. *putida* GN146) or 0.05 (*E*. *coli*) and incubated at 30°C or 37°C and 200 rpm in Erlenmeyer flasks. When the OD_600_ reached a value of 0.2, the cultures were induced (inducers: 0.2% (w/v) D-mannitol, 0.2% (w/v) D-arabitol, 0.2% (w/v) D-glucitol, 0.2% (w/v) L-rhamnose, 400 ng ml^-1^ anhydrotetracycline). A parallel culture was left uninduced as control. For fluorescence measurement, the cultures were diluted with the medium used for cultivation to an OD_600_ of 0.1. The fluorescence (485 nm excitation wavelength, 535 nm emission wavelength) of 100 μl of the diluted cultures was measured by a Genios microplate reader in fluorescence top measurement mode (Tecan, Crailsheim, Germany). The fluorescence of 100 μl culture medium was also measured and the obtained value was subtracted from the fluorescence value of the culture samples. An OD_600_ of 1 corresponds to 1×10^9^ cells.

### Transcription start site identification of P_*mtlE*_

Identification of the transcription start site of P_*mtlE*_ was performed by a modified 5’-RACE (rapid amplification of cDNA ends) method as described by Wang *et al*. [46]. *P*. *putida* GN146 was transformed with pJOE7771.1 by electroporation. 5 ml LB with 50 μg ml^-1^ kanamycin were inoculated with a single colony of *P*. *putida* pJOE7771.1 and incubated overnight at 30°C and 200 rpm. Next, 100 ml LB with 50 μg ml^-1^ kanamycin were inoculated with the overnight culture starting from an OD_600_ of 0.07 and incubated at 30°C and 200 rpm. After 2 h, 1% (w/v) D-mannitol was added and the culture was further incubated for 4 h at 30°C and 200 rpm. 1×10^9^ cells were harvested by centrifugation (5 min, 16,000× *g*) and total mRNA was extracted with the RNeasy Mini Kit (Qiagen, Hilden, Germany) according to the manufacturer´s protocol.

cDNA was synthesized at 42°C for 60 min in a total reaction volume of 20 μl containing 1 μg RNA, 1 μM oligonucleotide S8398, 40 U reverse transcriptase AMV (Roche, Mannheim, Germany), 25 U RNase inhibitor, 0.2 mM dNTPs, and 2 μl 10× reaction buffer for reverse transcriptase AMV. RNA was degraded by addition of 10 μl 2 N NaOH and incubation at 65°C for 30 min. 20 μl 1 N HCl were added for neutralization.

The cDNA was purified and ligated overnight at room temperature with the T4 RNA ligase (Thermo Scientific, Dreieich, Germany) in a total volume of 20 μl containing 13 ng cDNA, 10 U T4 RNA ligase, 2 μl 10× reaction buffer for T4 RNA ligase, and 0.1 mg ml^-1^ BSA. 20 ng of purified ligated cDNA were applied as a template for PCR in a total reaction volume of 50 μl containing 1 μM S8399, 1 μM S8400, 0.2 mM dNTPs, 1 U Phusion Hot Start DNA polymerase (Thermo Scientific, Dreieich, Germany), and 10 μl 5× reaction buffer for Phusion Hot Start DNA polymerase.

The obtained PCR product was purified and ligated with the 2,831 bp *Eco*RV fragment of pJOE4786.1. *E*. *coli* JM109 was transformed with the ligated fragments and correct plasmids were identified by restriction digest with *Nde*I. Four plasmids were selected (pJH165 plasmids, Table 1) and sequenced with oligonucleotide T7 for determination of the transcription start site of P_*mtlE*_.

### Purification of MtlR by affinity chromatography

The poor solubility of AraC/XylS transcriptional regulators is a known problem that often hinders *in vitro* studies of these proteins [21,24,47,48]. Making no exception, most of MtlR was also located in the insoluble fraction when *mtlR* was overexpressed in *E*. *coli* HB101 (S1 Fig). Although no soluble MtlR was detectable in the induced crude extract by SDS-PAGE, the low amounts that were present could be enriched by affinity chromatography yielding enough purified MtlR for *in vitro* studies (S1 Fig). A preliminary *in vivo* test was performed for determination of the optimal position of the *Strep*-tag II. First, *mtlR* was deleted from the sequence of pJOE7771.1 (yielding pJH189.1). The gene was separately amplified by PCR and subsequently integrated into three different pBR322-based vectors (see plasmid construction) allowing rhamnose-inducible synthesis of MtlR with an N-terminal *Strep*-tag II (pJH175.1), MtlR without tag (pJH176.2), or MtlR with a C-terminal *Strep*-tag II (pJH204.1). *E*. *coli* HB101 was transformed with pJH189.1 or pJH189.1 together with pJH175.1, pJH176.2, or pJH204.1, respectively, induced with mannitol and/or rhamnose, and the fluorescence was measured for quantification of P_*mtlE*_ activity (Table 2, S2 Fig). MtlR with a C-terminal *Strep*-tag II tag was able to induce *eGFP* expression comparably to the wild type MtlR without tag. Therefore, the *mtlR* gene (906 bp) of *P*. *fluorescens* DSM 50106 was expressed as a C-terminal *Step*-tag II fusion protein (35,960 Da) with *E*. *coli* HB101 pJH204.1. Cultivation, induction, cell disruption, and purification with 1 ml *Strep*-Tactin Sepharose were performed as described by Hoffmann *et al*. [44]. Buffer W (50 mM Tris-HCl pH 7.0, 50 mM NaCl, 100 mM KCl) was used for cell disruption and washing of the column, buffer E (buffer W with 2.5 mM *d*-desthiobiotin) was used for elution of the recombinant protein. Purification typically resulted in 400–500 μg ml^-1^ purified MtlR in the third elution fraction (500 μl). Uninduced and induced samples were analysed by SDS-PAGE (S1 Fig).

**Table 2:** Relative fluorescence of plasmid-carrying *E*. *coli* HB101 or *P*. *putida* GN146 strains measured 6 h after inducer addition. Unless stated otherwise, cultures were induced with mannitol.

Plasmid	Relative fluorescence of 1×10^7^ cells		Induction ratio
	uninduced	induced	
**Comparison of different promoter/regulator systems and inducers (Fig 3)**			
**pJOE7771.1^*a*^ (MtlR/P_*mtlE*_, mannitol)**	222 ± 46	4883 ± 419	22.0
**pJOE7771.1^*a*^ (MtlR/P_*mtlE*_, arabitol)**	222 ± 46	4363 ± 210	19.7
**pJOE7771.1^*a*^ (MtlR/P_*mtlE*_, glucitol)**	222 ± 46	1904 ± 147	8.6
**pJOE7784.1^*a*^ (RhaR-RhaS/P_*rhaBAD*_, rhamnose)**	66 ± 17	4702 ± 237	71.2
**pJOE7801.1^*a*^ (TetR/P_*tetA*_, anhydrotetracycline)**	94 ± 37	3605 ± 484	38.4
**P_*mtlE*_ mutants with altered -35 sequence “TTGTCA” (Fig 3B)**			
**pJH256.1^*a*^ (agGTCg)**	-116 ± 88	82 ± 67	n/a
**pJH257.2^*a*^ (TTGTCg)**	22 ± 7	3881 ± 181	176.4
**pJH258.1^*a*^ (TgGTCg)**	-38 ± 58	1396 ± 236	n/a
**Mutants with truncated sequences 5’ to P_*mtlE*_ (Fig 4A)**			
**pJH215.1^*a*^**	227 ± 84	4922 ± 827	21.7
**pJH219.1^*a*^**	256 ± 82	4892 ± 483	19.1
**pJH220.1^*a*^**	239 ± 34	4682 ± 443	19.61
**pJH228.1^*a*^**	320 ± 136	3598 ± 197	11.2
**pJH229.1^*a*^**	106 ± 76	937 ± 96	8.8
**pJH216.1^*a*^**	131 ± 65	576 ± 113	4.4
**pJH217.1^*a*^**	59 ± 36	118 ± 32	2.0
**pJH218.1^*a*^**	27 ± 30	71 ± 25	2.6
**pJH210.1^*a*^**	116 ± 3	121 ± 41	1.0
**Mutants with blocks of base substitutions 5’ to P_*mtlE*_ (Fig 4B)**			
**pJH221.1^*a*^**	117 ± 40	786 ± 58	6.7
**pJH222.1^*a*^**	54 ± 57	2113 ± 244	39.1
**pJH223.1^*a*^**	137 ± 48	108 ± 52	0.8
**pJH224.1^*a*^**	108 ± 57	337 ± 85	3.1
**pJH225.1^*a*^**	284 ± 158	3780 ± 567	13.3
**pJH226.1^*a*^**	233 ± 157	1357 ± 214	5.8
**pJH233.1^*a*^**	101 ± 43	581 ± 29	5.8
**pJH234.1^*a*^**	84 ± 69	105 ± 53	1.3
**pJH227.1^*a*^**	352 ± 142	4434 ± 541	12.6
**pJH230.1^*a*^**	198 ± 83	690 ± 52	3.5
**Mutants with doubled or shifted 15 bp sequence stretch -72 to -58 (Fig 4C)**			
**pJH253.7^*a*^**	3590 ± 74	3787 ± 233	1.1
**pJH255.1^*a*^**	3234 ± 256	3365 ± 330	1.0
**Effect of *Strep*-tag II fusions on the activity of MtlR (S2 Fig)**			
**pJH189.1^*a*^ (P_*mtlE*_-*eGFP*)**	2151 ± 257	2235 ± 177	1.0
**pJH189.1^*b*^(P_*mtlE*_-*eGFP*)**	2011 ± 323	1724 ± 71	0.9
**pJH189.1/pJH175.1^*b*^(P_*rhaBAD*_-*Strep*-tag II-*mtlR*)**	1737 ± 283	5241 ± 557	3.0
**pJH189.1/pJH176.2^*b*^(P_*rhaBAD*_-*mtlR*)**	1715 ± 126	6973 ± 385	4.1
**pJH189.1/pJH204.1^*b*^(P_*rhaBAD*_-*mtlR*-*Strep*-tag II)**	1818 ± 311	7178 ± 753	4.0

^*a*^ *P*. *putida* GN146.

^*b*^ *E*. *coli* HB101.

### Electrophoretic mobility shift assay (EMSA)

Cy5- or FITC- labelled operator fragments (S2 Table) were synthesized by PCR as described above with 5’-Cy5-labelled oligonucleotide S8533 or 5’-FITC-labelled oligonucleotide S10272, unlabelled oligonucleotide S8534 or S8485, and 0.4 ng of the P_*mtlE*_ operator mutant plasmids (Table 1) as template. DNA binding reactions were performed in a total volume of 25 μl containing 2 nM Cy5-labelled (8 nM FITC-labelled) PCR fragments, 445 nM (1,780 nM for FITC-labelled fragments) purified MtlR or 10 μl crude extract, and 5 μl 5× shift buffer A (50 mM Tris-HCl pH 7.0, 25% (v/v) glycerol, 5 mM tris(2-carboxyethyl)phosphine (TCEP), 250 μg ml^-1^ salmon sperm DNA, 20% (v/v) triethylene glycol (TEG), 10 mM D-mannitol). Samples were incubated for 30 min on ice. Subsequent electrophoresis was performed with 8.5 × 9 cm non-denaturing polyacrylamide gels (2.6 ml 30% (w/v) acrylamide/bisacrylamide (37.5:1), 2 ml 5× TBE (445 mM Tris base, 445 mM boric acid, 10 mM EDTA), 70 μl 10% (w/v) ammonium persulfate, 2 ml TEG, 3.3 ml H_2_O, 3.5 μl tetramethylethylenediamine). The gels were incubated at 42°C for 30 min for polymerization. Electrophoresis was carried out in 1× TBE at 6°C in a vertical electrophoresis system. The gels were equilibrated at 4–5 mA per gel for 10 min. The sample wells were flushed with running buffer immediately before 10 μl of the samples were applied. Separation of the samples was carried out at 10 mA per gel. The gels were scanned with the Storm 860 PhosphorImager (GE Healthcare, München, Germany).

### DNase I footprinting

EMSA reactions with a total volume of 200 μl containing 3 nM Cy5-labelled operator fragments (PCR 272, 273, 294, 295, 296, or 297, S2 Table), different concentrations of purified MtlR (70, 139, or 278 nM), and 40 μl 5× shift buffer A (see above) were incubated for 30 min on ice. Next, 10 μl were loaded on a shift gel and analysed as described above. The residual 190 μl of the reactions were mixed with 25 μl 100 mM MgCl_2_ and filled to 250 μl with H_2_O (resulting in 2.28 nM Cy5-labelled DNA and 66, 132, or 264 nM MtlR). Samples were incubated at room temperature for 15 min. Then 1 U (for digestion of PCR 272) or 0.57 U (for digestion of PCR 273) DNase I were added and the samples were incubated for 1 min at room temperature. Reactions were stopped by addition of 250 μl stop solution (50 mM EDTA, 15 μg ml^-1^ calf thymus DNA) and extracted with 500 μl phenol:chloroform: isoamyl alcohol 25:24:1. 400 μl of the supernatants were mixed with 800 μl 99.8% (v/v) ethanol and incubated at -70°C overnight. The DNA was pelleted by centrifugation (30 min, 16,000× *g*), washed with 500 μl 99.8% (v/v) ethanol, dried, resolved in 10 μl H_2_O, and mixed with 5 μl loading buffer (Affymetrix, High Wycombe, UK, see below). After heating for 2 min at 70°C, 5 μl of the samples were loaded onto a 0.3 mm thick sequencing gel containing 6% (w/v) acrylamide/bisacrylamide and 7.5 M urea (Rotiphorese Sequencing Gel System A431, Carl Roth, Karlsruhe, Germany). Sequencing reactions of pJOE7771.1, pJH253.7, and pJH255.1 were performed with oligonucleotides S9711 or S8533 and the Thermo Sequenase Cycle Sequencing Kit according to the manufacturer´s protocol (Affymetrix, High Wycombe, UK). Electrophoresis and analyses were performed with ALFexpress II DNA sequencer (formerly Amersham Pharmacia Biotech, Piscataway, NJ, USA). The experiments were independently repeated at least three times.

### Determination of the equilibrium dissociation constant and the dissociation rate of the MtlR-operator complex

Determination of the equilibrium dissociation constant *K*_D_ and the dissociation rate *k*_diss_ was performed as described by Rother *et al*. [49]. For determination of the equilibrium dissociation constant *K*_D_, EMSA reactions with total volumes of 25 μl containing 2 nM Cy5-labelled PCR fragment (PCR 210, S2 Table), different concentrations (2, 11, 56, 111, 167, 223, 445, or 667 nM) of purified MtlR, and shift buffer A (see above) or shift buffer B (shift buffer A without D-mannitol) were performed. Band intensities were determined with the ImageQuant 5.0 Software (formerly Amersham Pharmacia Biotech, Piscataway, NJ, USA). The amount of bound and free DNA was determined and plotted against the amount of purified MtlR used in the assay. The *K*_D_ values were taken from the plot.

Determination of the dissociation rate *k*_diss_ and the half-life time t_½_ of the MtlR-operator complex were also performed by EMSA. 100 μl reactions containing 2 nM Cy5-labelled operator fragment (PCR 210, S2 Table) and 445 nM purified MtlR were incubated on ice for 30 min. Dissociation of the MtlR-operator complex was initiated by addition of 100 nM non-labelled competitor DNA (PCR 211, S2 Table). 10 μl samples were taken in 15 min intervals and loaded onto a non-denaturing polyacrylamide gel with 20% (v/v) TEG (see above). Band intensities were determined with the ImageQuant 5.0 Software. The amount of bound DNA was determined for each lane. The equation ln([DNA-MtlR]_t_/[DNA-MtlR]_t0_) = –*k*_diss_t was used for analysis of the binding data. [DNA-MtlR]_t_ is the concentration of the MtlR-operator complex at time t and [DNA-MtlR]_t0_ is the concentration of the complex immediately after addition of the competing DNA. The obtained data were plotted against the time. The slope of the linear regression equals–*k*_diss_. The half-life time of the MtlR-operator complex was calculated with the equation t_½_ = ln2/*k*_diss_. All experiments were repeated at least three times.

## Results

### Construction and optimization of an *mtlR*/P_*mtlE*_ expression vector

*mtlR* and the *mtlE-Z* operon are located separately from each other in the genome of *P*. *fluorescens* DSM50106. In order to construct an expression vector containing the functional *mtlR*/P_*mtlE*_ regulatory unit, P_*mtlE*_ was fused to the reporter gene *eGFP* and integrated into a pBBR1MCS-2-based vector. The *mtlR* gene under control of its wild type promoter was inserted upstream of P_*mtlE*_ in opposite orientation yielding pJOE7771.1 (Fig 2, see materials and methods for details on construction). The transcription start site of P_*mtlE*_ was identified by a modified 5’-RACE protocol (Fig 2 and materials and methods).

**Fig 3:**
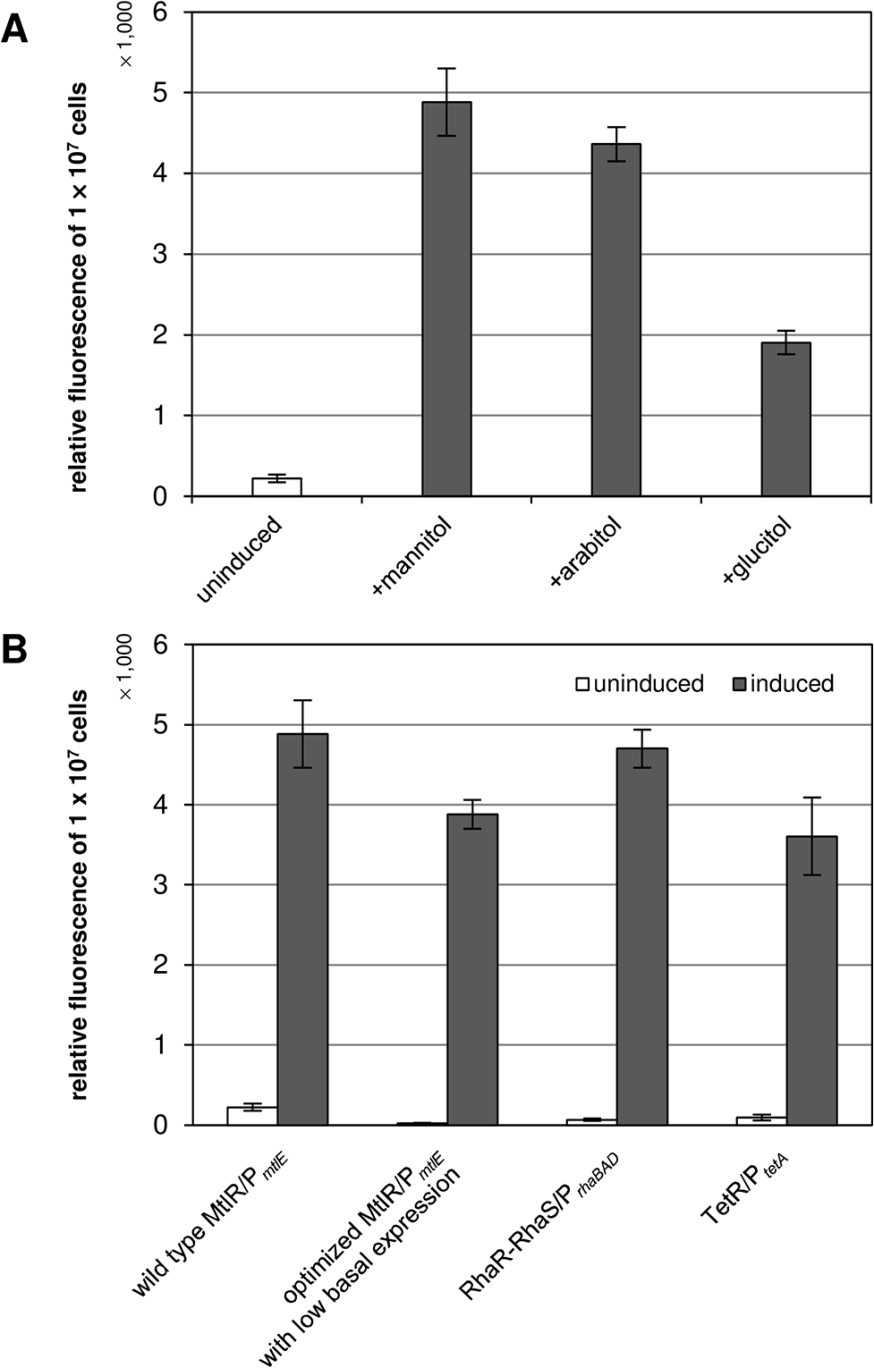
*eGFP* reporter gene expression with different regulator/promoter systems and inducers in *P*. *putida* GN146. (A) Fluorescence of *P*. *putida* GN146 pJOE7771.1 (MtlR/P_*mtlE*_) induced with mannitol, arabitol, or glucitol (B) Fluorescence of *P*. *putida* GN146 pJOE7771.1 (MtlR/P_*mtlE*_, inducer: mannitol) and pJH257.2 (optimized MtlR/P_*mtlE*_ with altered -35 sequence “TTGTCg”, inducer: mannitol) compared to *P*. *putida* GN146 pJOE7784.1 (RhaR-RhaS/P_*rhaBAD*_, inducer: rhamnose) and *P*. *putida* GN146 pJOE7801.1 (TetR/P_*tetA*_, inducer: anydrotetracycline). Fluorescence was measured 6 h after inducer addition.

Expression of *eGFP* was investigated with *P*. *putida* GN146 pJOE7771.1 with or without mannitol, arabitol, or glucitol, respectively and the obtained fluorescence data were compared to those of the well-known RhaR-RhaS/P_*rhaBAD*_ [32,50] and TetR/P_*tetA*_ (see materials and methods) expression systems. Fluorescence was measured 6 h after addition of inducers. Mannitol and arabitol induced *eGFP* expression from P_*mtlE*_ comparably, while the induction ratio with glucitol was about 2.5-fold lower (Fig 3A, Table 2). The mannitol-induced expression level of the MtlR/P_*mtlE*_ system was higher than that of the TetR/P_*tetA*_ expression system and comparable to the RhaR-RhaS/P_*rhaBAD*_ system, albeit with 3.4-fold higher basal expression (Fig 3B, Table 2). In order to lower the basal expression from P_*mtlE*_, three different plasmids with altered -35 sequences were constructed. The -35 sequence “TTGTCA” of the wild type was changed to “agGTCg” (pJH256.1), “TTGTCg” (pJH257.2), or “TgGTCg (pJH258.1) (Table 1). The best result was obtained with pJH257.2. The mutant is characterized by strongly reduced basal expression (10-fold lower compared to the wild type) and only slightly (1.3-fold) reduced activity of mannitol-induced P_*mtlE*_ leading to a highly increased induction ratio compared to the wild type (Table 2, Fig 3B).

### *In vivo* analysis of the MtlR binding site

**Fig 4:**
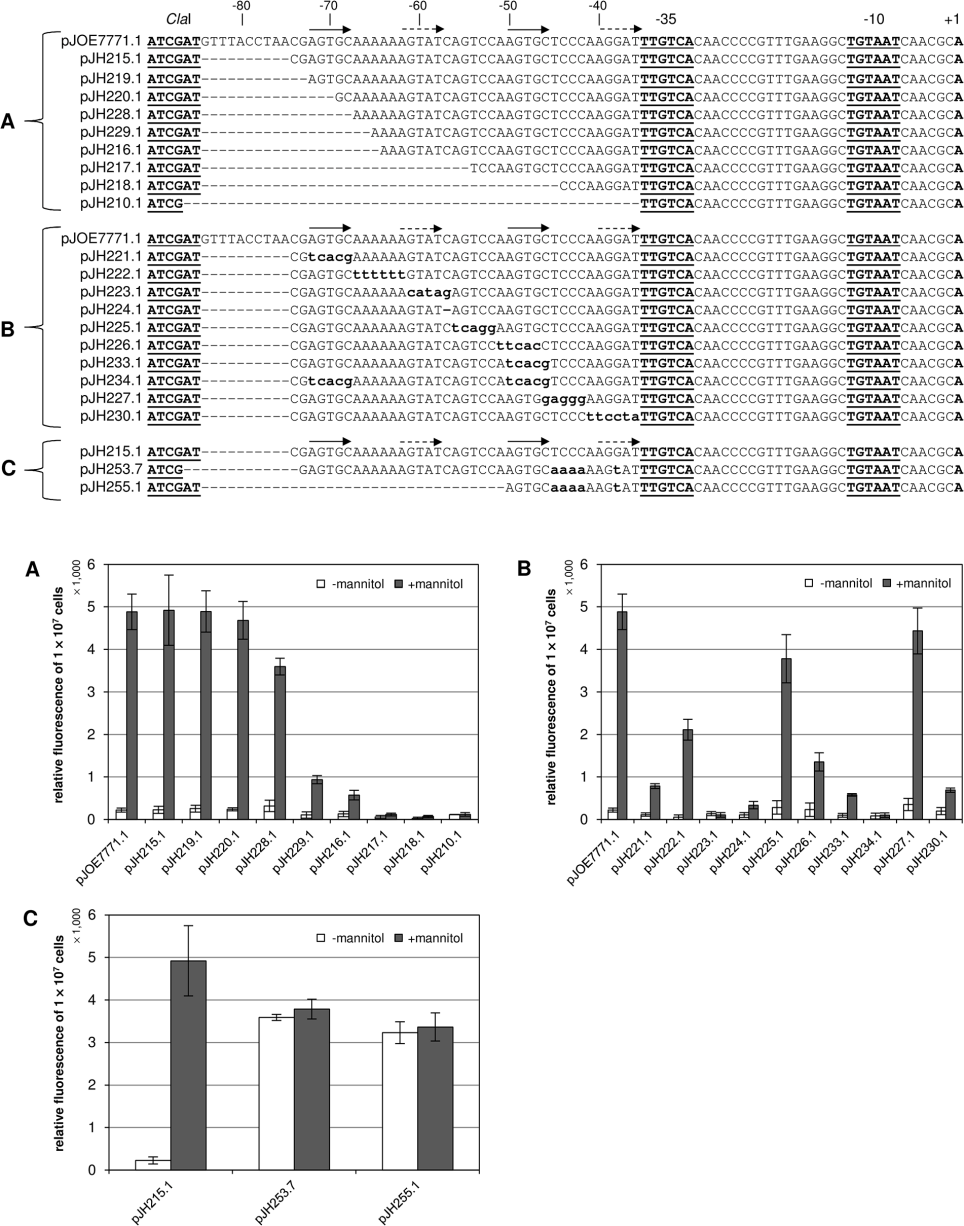
*In vivo* analysis of the MtlR binding site by fluorescence measurement of *P*. *putida* GN146 strains carrying pJOE7771.1-derived mutant plasmids. The nucleotide sequences of the wild type (pJOE7771.1) and the mutants are shown. Mutated nucleotides are typed in lowercase. Perfect direct repeats are indicated by solid arrows. Similar direct repeats are indicated by dashed arrows. Fluorescence was measured 6 h after addition of mannitol. (A) Mutants with truncated 5’ sequences. (B) Mutants with blocks of base substitutions. (C) Mutants with doubled or shifted 15 bp sequence stretch -72 to -58.

Sequence analysis of the 5’ region of P_*mtlE*_ revealed two perfect and two similar direct repeats (indicated by solid and dashed arrows) and also a striking poly A-tract adjacent to the first repeat (Fig 4). In order to analyse whether these structural elements are involved in P_*mtlE*_ activation and to identify the MtlR binding site, several pJOE7771.1-derived mutant plasmids with truncated or otherwise mutated sequences located 5’ to the transcription start site were constructed and P_*mtlE*_ activity was quantified by fluorescence measurement of plasmid-carrying *P*. *putida* GN146 strains.

Stepwise truncation of the 5’ sequence of P_*mtlE*_ revealed a strong decrease in promoter activity and induction ratio when less than 67 base pairs of the original sequence were present 5’ to the transcription start site (pJH229.1, Fig 4A, Table 2). At least 69 base pairs upstream of the transcription start site were necessary to sustain wild type P_*mtlE*_ activity (pJH220.1). Hence, P_*mtlE*_ transcription activation was not affected when the first two base pairs of the first repeat were deleted (note that the “T” of the *Cla*I site of pJH220.1 complements deletion of the “T” of the first repeat).

Next, starting from the -72 position (start of the first repeat), blocks of 5 bp length were replaced by their complementary sequences and P_*mtlE*_ activity was measured in plasmid-carrying *P*. *putida* GN146 strains as before (Fig 4B, Table 2). Mutation of the first 5 bp (the complete first repeat) strongly decreased P_*mtlE*_ activity and induction ratio (pJH221.1). Mutation of the second repeat also had a severe effect on P_*mtlE*_ activity (pJH226.1, pJH233.1) and when both repeats were mutated, the negative effect on P_*mtlE*_ was even more pronounced (pJH234.1). Substitution of base pairs -57 to -61 or deletion of the “C” at position -57 also abolished P_*mtlE*_ inducibility (pJH223.1, pJH224.1). Substitution of the 5 base pairs immediately upstream of the -35 sequence had a similar effect (pJH230.1). Some mutations affected P_*mtlE*_ activity to a lesser extent. Replacement of the poly A stretch following the first repeat by a poly T sequence (base pairs -62 to -67, pJH222.1) reduced the induced expression level of P_*mtlE*_ 2.3-fold. However, as an exception to all other measured operator mutants, the very low basal activity of this construct resulted in a higher induction ratio than the wild type (Table 2). Substitution of base pairs -52 to -56 (pJH225.1) and -42 to -46 (pJH227.1) by their complementary base pairs slightly reduced the induction ratio.

P_*mtlE*_ was rendered constitutive when the sequence between bp -50 and -36 was adjusted to the sequence between bp -72 and -58 resulting in two identical 15 bp sequence stretches including the indicated direct repeats (pJH253.7, Fig 4C, Table 2). Interestingly, constitutivity was retained when only the altered downstream sequence was present (pJH255.1).

The presented results indicate that the MtlR binding site stretches out over at least 37 base pairs located upstream of the -35 sequence of P_*mtlE*_. Two similar but not identical 15 bp long blocks containing direct repeats seem to be particularly involved in mannitol-dependent activation of P_*mtlE*_.

### *In vitr*o analysis of the MtlR binding site by EMSA and DNase I footprinting experiments

The *in vivo* experiments with *P*. *putida* GN146 and the mutant plasmids revealed the effect of the operator mutations on the activity of P_*mtlE*_ but their effect on DNA binding by MtlR was still unclear. The DNA binding properties of MtlR were studied by EMSA, a common method for characterization of DNA binding proteins. For this purpose, purified MtlR (see materials and methods) or induced crude extracts of *E*. *coli* HB101 pJH204.1 were incubated with Cy5-labelled operator DNA and analysed by EMSA following a standard protocol. Extensive band smearing was observed, when MtlR was present in the sample (S3A Fig). This was an evidence for occurrence of DNA binding by MtlR but the DNA-protein complex seemed to be very unstable dissociating either when the sample was loaded onto the gel or during electrophoresis. Several experiments were carried out varying pH value, ion strength of the buffer, temperature, additives like MgCl_2_, KCl, salmon sperm DNA, mannitol, fructose, fructose 6-phosphate, glucitol, or arabitol but none of them gave feasible results (data not shown). It has been described in literature that triethylene glycol (TEG) can stabilize labile DNA-protein complexes in polyacrylamide gels [51]. The addition of TEG actually enhanced the stability of the MtlR-operator complex and thus enabled detailed analysis of the DNA binding properties of MtlR (S3B Fig).

**Fig 5:**
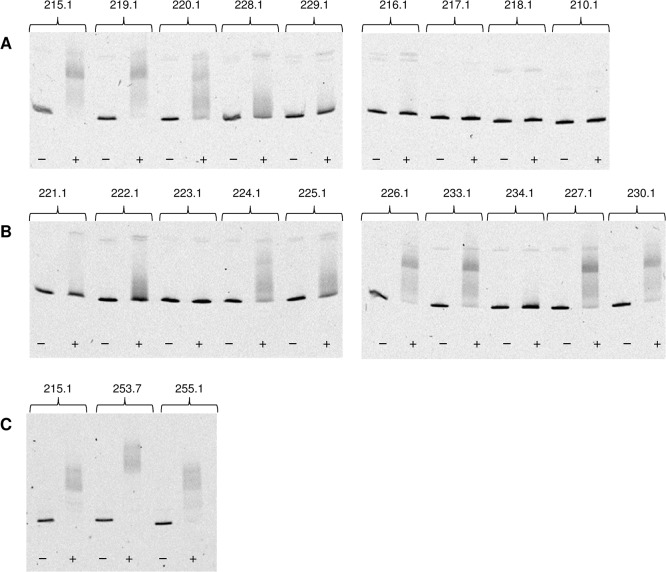
EMSA of 2 nM Cy5-labelled (or 8 nM FITC-labelled) DNA fragments incubated (+) with 445 nM (or 1,780 nM for FITC-labelled fragments) or (-) without MtlR. (A) P_*mtlE*_ operator mutants with truncated 5’ sequences (fragments Cy5-labelled). (B) P_*mtlE*_ operator mutants with blocks of base substitutions (fragments Cy5-labelled). (C) P_*mtlE*_ operator mutants with doubled or shifted 15 bp sequence stretch -72 to -58 (fragments FITC-labelled). The numbers of the DNA fragments equal the numbers of the plasmids in Fig 4.

Cy5- or FITC-labelled DNA fragments containing the relevant nucleotide sequences of the plasmids used for the *in vivo* studies (Fig 4, S2 Table) were incubated with purified MtlR and analysed by EMSA (Fig 5). Deletion of base pairs -73 and -74 did not influence DNA binding by MtlR (DNA fragment 219.1, Fig 5A). This result is in agreement with the fluorescence data obtained with *P*. *putida* GN146 pJH219.1 (Fig 4A). In contrast, *P*. *putida* GN146 pJH220.1 showed P_*mtlE*_ activity comparable to the wild type (Fig 4A), but MtlR binding was clearly reduced *in vitro* (fragment 220.1, Fig 5A). Hence, although no effect on transcription activation could be measured *in vivo*, base pairs -71 and -72 were demonstrated to be important for DNA binding of MtlR *in vitro*. Further truncation of the sequence 5’ to P_*mtlE*_ gradually reduced MtlR binding (228.1–216.1, Fig 5A). When 54 or less original base pairs were left 5’ to the transcription start site, no binding of MtlR was observed (217.1–210.1, Fig 5A). The *in vitro* data obtained with DNA fragments 221.1, 223.1, 234.1, and 227.1 (Fig 5B) also reflected the fluorescence data obtained by the *in vivo* experiments (Fig 4B).

In some other cases, the *in vitro* and *in vivo* results did not match. Although weaker as compared to the control 215.1, DNA binding of MtlR to the fragment 224.1 was clearly visible by EMSA (comparable to fragment 220.1) but almost no transcription activation from P_*mtlE*_ was measured with *P*. *putida* GN146 pJH224.1 (Figs 5B and 4B). Binding of MtlR to the fragments 226.1, 233.1, and 230.1 was only slightly, if at all, reduced compared to the control fragment 215.1 (Fig 5B) but transcription activation from P_*mtlE*_ occurred disproportional weakly (*P*. *putida* GN146 pJH226.1, pJH233.1, and pJH230.1) (Fig 4B). In addition, transcription activation from P_*mtlE*_ was stronger in *P*. *putida* GN146 pJH226.1 than in *P*. *putida* GN146 pJH233.1 and pJH230.1 but no difference in DNA binding by MtlR was observable with EMSA. Likewise, the DNA fragments 222.1 and 225.1 shifted comparably, but the transcription activation from P_*mtlE*_ in *P*. *putida* GN146 pJH222.1 occurred weaker than in *P*. *putida* GN146 pJH225.1.

The *in vivo* experiments clearly demonstrated involvement of base pairs -50 to -46 and -41 to -36 in transcription activation from P_*mtlE*_ but this could not be confirmed by EMSA. These results appear contradictorily at first sight but can be brought in line when MtlR is considered to act as a dimer and when binding of only one monomer to the upstream binding site was detected by EMSA. If this was the case, binding of MtlR to the upstream binding site must be stronger than to the downstream binding site. Actually, a larger complex, likely representing the MtlR dimer, could be detected by EMSA when two of the strong binding sites were present (pJH253.7, Figs 4C and 5C). A smaller complex corresponding to one bound MtlR monomer was observed when the strong binding site was shifted towards the -35 sequence (pJH255.1, Figs 4C and 5C).

**Fig 6:**
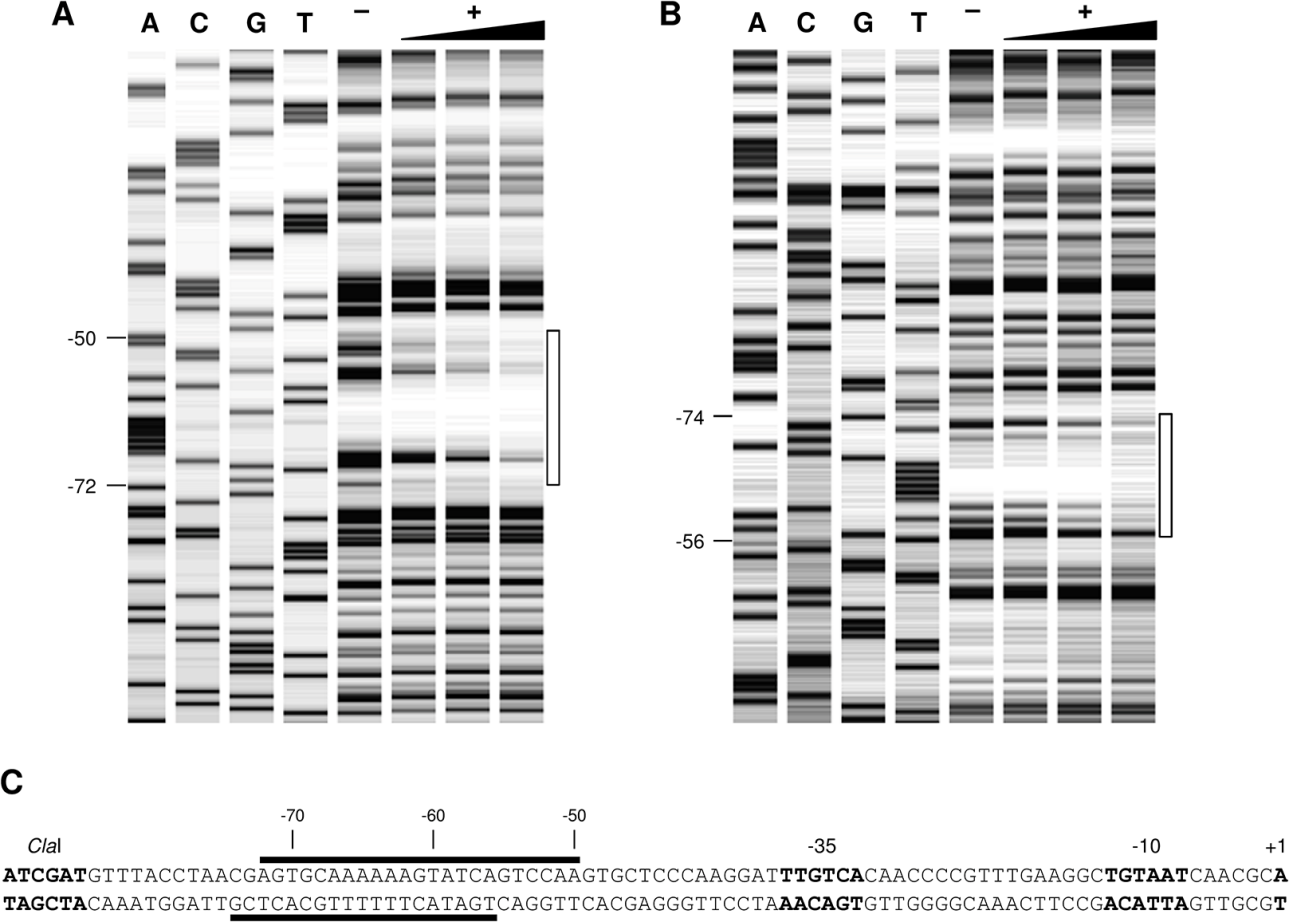
DNase I footprinting analysis of the MtlR binding site. One representative experiment is shown. The sequencing reaction (ACGT) of pJOE7771.1 is shown on the left. Footprinting reactions were performed with 2.28 nM Cy5-labelled operator DNA (-) without or (+) with MtlR (66, 132 or 264 nM). The protected nucleotides are indicated by empty rectangles on the right and the bases that mark the borders of the protected region are indicated on the left. (A) Coding strand. (B) Non-coding strand. (C) Presentation of the nucleotides protected by MtlR in the sequence 5’ to P_*mtlE*_ by black lines above (coding strand) and below (noncoding strand) the sequence.

Investigation of the MtlR binding site by DNase I footprinting experiments indicated 23 protected bases (base pairs -50 to -72) on the coding strand and 19 protected bases (base pairs -56 to -74) for the non-coding strand in the wild type sequence (Fig 6). This region corresponds to the postulated strong upstream binding site of MtlR. When two strong binding sites were present, 43 protected bases (base pairs -28 to -70) were found on the coding strand and 43 protected bases (base pairs -28 to -74) on the non-coding strand (S4 Fig). This corresponds to the region occupied by the MtlR dimer. When the strong binding site was shifted towards the -35 region, 22 (base pairs -29 to -50) and 25 (base pairs -30 to -54) protected bases were found on the coding strand and non-coding strand, respectively (S5 Fig).

**Fig 7:**
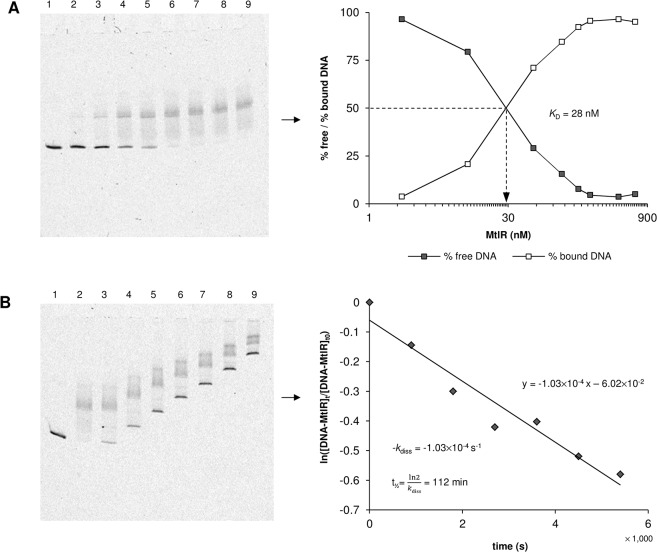
Determination of the equilibrium dissociation constant (*K*_*D*_), dissociation rate (*k*_*diss*_) and half life time (t_*½*_) of the MtlR monomer binding to its upstream binding site. (A) Representative EMSA and determination of *K*_*D*_. Lanes: (1) 2 nM Cy5-labelled operator DNA, (2–9) 2 nM Cy5-labelled operator DNA + 2, 11, 56, 111, 167, 223, 445, or 667 nM MtlR. The average *K*_*D*_ values of at least three independent experiments were 30.8 ± 4.8 nM with mannitol and 32.6 ± 5.0 nM without mannitol. (B) Representative EMSA and determination of *k*_*diss*_ and t_*½*_. Lanes: (1) 2 nM Cy5-labelled operator DNA, (2) 2 nM Cy5-labelled operator DNA + 445 nM MtlR, (3–9) 2 nM Cy5-labelled operator DNA + 445 nM MtlR + 100 nM non-labelled competitor DNA loaded onto the gel 0, 15, 30, 45, 60, 75, and 90 min after addition of the competitor. The average *k*_*diss*_ and t_*½*_ values of at least three independent experiments were 1.1×10^−4^ ± 2.8×10^−5^ s^-1^ and 112 ± 24 min with mannitol and 1.2×10^−4^ ± 2.8×10^−5^ s^-1^ and of 99 ± 23 min without mannitol. The second upper band in some of the lanes on gel B is considered as an electrophoresis artefact (compare Fig 5C).

The DNA binding properties of the MtlR monomer bound to the strong upstream binding site of the wild type sequence were determined (Fig 7). The obtained equilibrium dissociation constants *K*_D_ (defined as the concentration of MtlR that shifts 50% of the operator DNA) were 30.8 ± 4.8 nM with mannitol and 32.6 ± 5.0 nM without mannitol. The dissociation rates *k*_diss_ and the half life times t_½_ of the MtlR-operator complex were 1.1×10^−4^ ± 2.8×10^−5^ s^-1^ and 112 ± 24 min with mannitol and 1.2×10^−4^ ± 2.8×10^−5^ s^-1^ and 99 ± 23 min without mannitol.

The data suggest that MtlR functions as a dimer and that dimerization might occur upon DNA binding. Since P_*mtlE*_ becomes constitutive when the downstream binding site is adjusted to the strong upstream binding site, the second weaker binding site can be identified as a key component in the activation mechanism that underlies mannitol-dependent induction of P_*mtlE*_. Binding affinity of the MtlR monomer to the upstream binding site is not affected by mannitol.

## Discussion

A new pBBR1MCS-2-based expression vector containing the functional *mtlR*/P_*mtlE*_ regulatory unit from *P*. *fluorescens* DSM10506 was constructed and expression of the *eGFP* reporter gene was investigated with *P*. *putida* GN146. Reporter gene expression with the MtlR/P_*mtlE*_ system was comparable to those of RhaR-RhaS/P_*rhaBAD*_ and TetR/P_*tetA*_ based systems, however with slightly higher basal expression. An optimized vector with very low basal expression was created by altering the -35 sequence. We also tried to enhance inducible gene expression from P_*mtlE*_ by integrating the mannitol transporter (*mtlEFGK*) into the chromosome of *P*. *putida* GN146. However, this predominantly elevated basal expression (6.7-fold) and only slightly improved the induced expression level of P_*mtlE*_ (1.4-fold) (data not shown). A special feature of the new MtlR/P_*mtlE*_ expression vector is the possibility to induce gene expression with three different effectors; mannitol, arabitol, or glucitol. Glucitol induces gene expression 2.5-fold lower compared to mannitol and arabitol. Sometimes strong expression impairs cell growth or results in inclusion body formation. One strategy to overcome these problems is lowering gene expression level [52,53]. The fact that expression with the MtlR/P_*mtlE*_ system can be varied by applying different inducers might be a helpful option for modulation of gene expression of a particular gene. In summary, we provide a new and very versatile expression vector that might be especially suitable for fermentation applications, where the possibility to use the cheap inducer mannitol offers a much more economical production than employing systems that require for example very expensive rhamnose or highly toxic anhydrotetracycline. The fact that *P*. *putida* cannot utilize mannitol as a carbon source further reduces inducer consumption and thereby costs.

Another focus of this study was examination of the MtlR binding site. Two perfect and two similar direct repeats were found in the DNA sequence 5’ to the transcription start site of P_*mtlE*_ (Fig 4). The performed *in vivo* experiments as well as EMSA and DNaseI footprinting analyses confirmed that the first repeat actually marks the beginning of the MtlR binding site. As already mentioned, AraC/XylS type regulators involved in carbon metabolism normally form dimers [12–14,54]. An N-terminal AraC-like ligand binding and dimerization domain could also be found in the deduced amino acid sequence of MtlR (BLAST search with GenBank accession number AAC34292.1, data not shown). DNase I footprinting analysis of the wild type MtlR-operator complex revealed 23 protected bases on the coding strand and 19 protected bases on the non-coding strand. The protected sequence of the noncoding strand is probably a bit longer than detected because of the gap of DNase I fragments in the control (Fig 6B). In both cases, the protected sequence is too short for binding of a dimer because one AraC/XylS type DNA binding domain occupies about 15 base pairs [16–18]. On the other hand, the *in vivo* experiments clearly demonstrate that base pairs -46 to -49 and -36 to -40 that are not part of the DNaseI protected region are important for P_*mtlE*_ activation. Furthermore, the distance of the first repeat (the beginning of the MtlR binding site) to the -35 region of P_*mtlE*_ is as long as those of the bipartite XylS binding site to P_m_ and MekR binding site to P_*mekA*_ [15,20,21]. It has been described that the upstream binding half sites of the MelR and RhaS dimers are bound significantly more strongly than the downstream half sites [55,56]. This also seems to be the case for MtlR and explains the missing footprint of the second monomer. The MtlR dimer could be detected by EMSA and DNaseI footprinting when the sequence of the weaker downstream binding site was adjusted to the sequence of the upstream binding site. However, two strong binding sites or the presence of only one strong downstream binding site rendered P_*mtlE*_ constitutive. Consequently, the weaker downstream binding site is a key element for mannitol-dependent regulation of P_*mtlE*_. The totality of the data suggests that that MtlR binds to P_*mtlE*_ as a dimer with the consensus sequence AGTGC-N_5_-AGTAT-N_7_-AGTGC-N_5_-AGGAT.

The DNA-sequence 5’ to the transcription start site of P_*mtlE*_ includes a striking poly A-tract in the coding strand directly following the first repeat (Fig 4). The sequence stretch ‘TGCAAAAAA’ can also be found in the binding site of XylS but there it is located closer to the promoter being part of the binding motif of the second XylS monomer [15,19,20]. Poly A-tracts are widely distributed and well-studied DNA elements that are particularly located in regulatory regions (reviewed in [57]). They influence the curvature of the DNA due to their unique molecular structure that differs from the canonical B-DNA conformation. Replacement of the poly A stretch of the coding strand to a poly T sequence in the P_*mtlE*_ operator strongly reduced the basal activity of P_*mtlE*_ leading to a higher induction ratio compared to the wild type (Fig 4B, Fig 5B). This indicates that the poly A-tract is not only involved in stabilization of MtlR binding as suggested by the EMSA studies but might also influence the kinetic parameters of the multistep transcription initiation process including promoter recognition, open complex formation, and promoter clearance.

Transcription activation by AraC/XylS regulators is usually attributed to effector-induced structural changes that influence DNA binding affinity and/or sterical orientation of amino acid residues that contact subunits of the RNA polymerase. For example, XylR from *E*. *coli* does not bind to its target DNA without xylose. Binding of xylose to XylR induces a helix to strand transition in the N-terminal domain that increases DNA binding affinity of the XylR dimer [58]. In contrast, the experimental data obtained with RhaR and its effector rhamnose indicate, that an improved contact with subunits of the RNA polymerase is rather the basis for transcription activation than an increased DNA binding affinity [25]. The equilibrium dissociation constant and the dissociation rate of the MtlR monomer bound to its upstream half site were not affected by mannitol, indicating that binding of the MtlR monomer to the strong half site occurs independently from mannitol. Because the second monomer could not be detected by EMSA or DNaseI footprinting even in the presence of mannitol, it cannot be excluded that mannitol influences the DNA binding affinity of the second MtlR monomer binding to the weaker downstream half site. A possible explanation for the missing detection of the wild type dimer complex in our *in vitro* studies is potential involvement of the RNA polymerase in complex formation. The monomer binding to the weaker half site might be anchored by the second monomer on one side and the RNA polymerase on the other side. Mannitol might induce a conformational reorientation of MtlR that enables transcription activation. EMSA and *in vitro* transcription studies with RNA polymerase, purified MtlR and mutants thereof are appropriate approaches to further elucidate the specific mechanism underlying transcription activation of P_*mtlE*_ by MtlR.

## Supporting Information

S1 Fig SDS-PAGE analysis of crude extracts and *Strep*-tag II purification fractions of *E*. *coli* HB101 pJH204.1. (PDF)

S2 Fig Effect of *Strep*-tag II fusions on the activity of MtlR. (PDF)

S3 Fig Electrophoretic mobility shift assays (EMSA) of crude extracts of *E*. *coli* HB101 pJH204.1 or purified MtlR incubated with Cy5-labelled DNA fragments. (PDF)

S4 Fig DNase I footprinting analysis of the mutated MtlR binding site of pJH253.7. (PDF)

S5 Fig DNase I footprinting analysis of the mutated MtlR binding site of pJH255.1. (PDF)

S1 Table Oligonucleotides used in this study. (PDF)

S2 Table PCRs used for EMSA and DNaseI Footprinting experiments. (PDF)
