# Supplementary material for: Functional Characterization of the Mannitol Promoter of Pseudomonas fluorescens DSM 50106 and Its Application for a Mannitol-Inducible Expression System for Pseudomonas putida KT2440
Source: PLoS One. 2015 Jul 24;10(7):e0133248. doi: 10.1371/journal.pone.0133248 (PMC4514859; doi:10.1371/journal.pone.0133248)
Supplement: S1 Fig — (PDF) [file pone.0133248.s001.pdf]

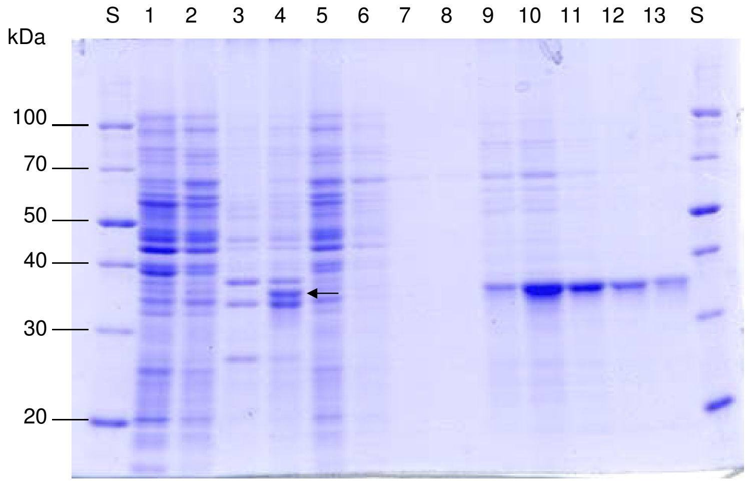

Fig S1. SDS-PAGE analysis of crude extracts and *Strep*-tag II purification fractions of *E. coli* HB101 pJH204.1. Lanes: (S) molecular weight marker, (1) uninduced crude extract, soluble fraction, (2) induced crude extract, soluble fraction, (3) uninduced crude extract, insoluble fraction, (4) induced crude extract, insoluble fraction, (5) flow through fraction, (6) second washing fraction, (7) fifth washing fraction, (8-13) elution fractions 1-6. Insoluble MtlR is marked by an arrow.
