# Supplementary material for: Functional Characterization of the Mannitol Promoter of Pseudomonas fluorescens DSM 50106 and Its Application for a Mannitol-Inducible Expression System for Pseudomonas putida KT2440
Source: PLoS One. 2015 Jul 24;10(7):e0133248. doi: 10.1371/journal.pone.0133248 (PMC4514859; doi:10.1371/journal.pone.0133248)
Supplement: S2 Fig — (PDF) [file pone.0133248.s002.pdf]

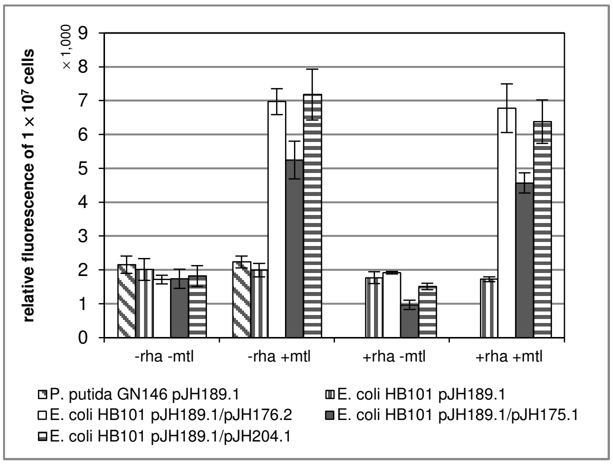

Fig S2. Effect of *Strep*-tag II fusions on the activity of MtlR. The fluorescence of *P. putida* GN146 pJH189.1 or *E. coli* HB101 carrying compatible plasmids pJH189.1 (P_{mtlE}-*eGFP*) together with pJH175.1 (P_{rhaBAD}-*Strep*-tag II-*mtlR*), pJH176.2 (P_{rhaBAD}-*mtlR*), or pJH204.1 (P_{rhaBAD}-*mtlR-Strep*-tag II) is shown. Fluorescence was measured 6 h after inducer addition, rha = rhamnose, mtl = mannitol.
