# Supplementary material for: Functional Characterization of the Mannitol Promoter of Pseudomonas fluorescens DSM 50106 and Its Application for a Mannitol-Inducible Expression System for Pseudomonas putida KT2440
Source: PLoS One. 2015 Jul 24;10(7):e0133248. doi: 10.1371/journal.pone.0133248 (PMC4514859; doi:10.1371/journal.pone.0133248)
Supplement: S3 Fig — (PDF) [file pone.0133248.s003.pdf]

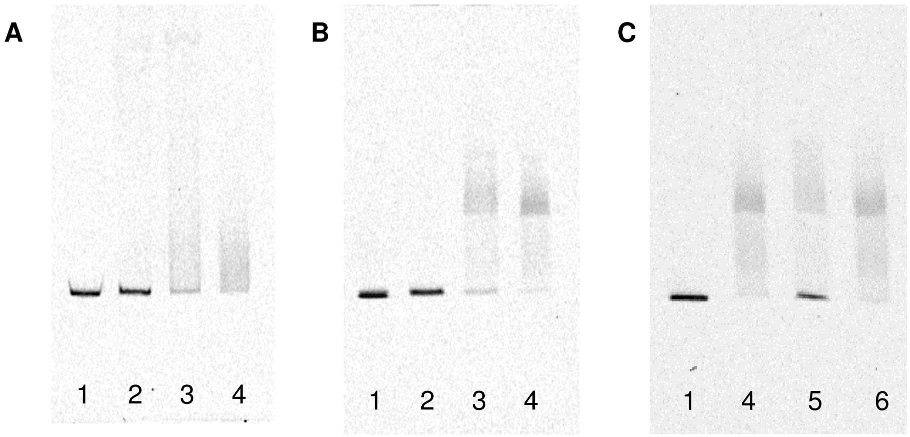

Fig S3. Electrophoretic mobility shift assays (EMSA) of crude extracts of *E. coli* HB101 pJH204.1 or purified MtlR incubated with Cy5-labelled DNA fragments. (A) Gel without triethylene glycol (TEG). (B) Gel with 20% (v/v) TEG. (C) Gel with 20% (v/v) TEG. Lanes: (1) 2 nM Cy5-labelled operator DNA, (2) 2 nM Cy5-labelled operator DNA + uninduced crude extract, (3) 2 nM Cy5-labelled operator DNA + induced crude extract, (4) 2 nM Cy5-labelled operator DNA + 445 nM MtlR, (5) 2 nM Cy5-labelled operator DNA + 445 nM MtlR + 100 nM unlabelled competitor DNA with MtlR binding site (PCR 211), sample loaded onto the gel immediately after addition of PCR 211, (6) 2 nM Cy5-labelled operator DNA + 445 nM MtlR + 100 nM unlabelled DNA without MtlR binding site (PCR 271), sample loaded on gel immediately after addition of PCR 271.
