# Supplementary material for: Functional Characterization of the Mannitol Promoter of Pseudomonas fluorescens DSM 50106 and Its Application for a Mannitol-Inducible Expression System for Pseudomonas putida KT2440
Source: PLoS One. 2015 Jul 24;10(7):e0133248. doi: 10.1371/journal.pone.0133248 (PMC4514859; doi:10.1371/journal.pone.0133248)
Supplement: S4 Fig — (PDF) [file pone.0133248.s004.pdf]

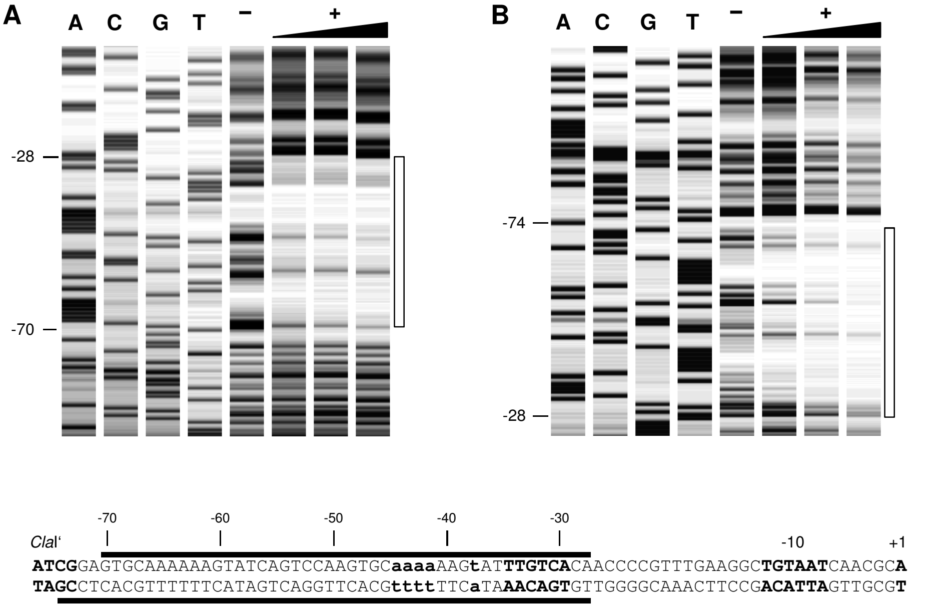

Fig S4. DNase I footprinting analysis of the mutated MtlR binding site of pJH253.7. One representative experiment is shown. The sequencing reaction (ACGT) of pJH253.7 is shown on the left. Footprinting reactions were performed with 2.28 nM Cy5-labelled operator DNA (-) without or (+) with MtlR (66, 132 or 264 nM). The protected nucleotides are indicated by empty rectangles on the right and the bases that mark the borders of the protected region are indicated on the left. (A) Coding strand. (B) Non-coding strand. (C) Presentation of the nucleotides protected by MtlR in the sequence 5' to P_{mtlE} by black lines above (coding strand) and below (noncoding strand) the sequence.
