# Supplementary material for: Functional Characterization of the Mannitol Promoter of Pseudomonas fluorescens DSM 50106 and Its Application for a Mannitol-Inducible Expression System for Pseudomonas putida KT2440
Source: PLoS One. 2015 Jul 24;10(7):e0133248. doi: 10.1371/journal.pone.0133248 (PMC4514859; doi:10.1371/journal.pone.0133248)
Supplement: S1 Table — (PDF) [file pone.0133248.s006.pdf]

TABLE S1. Oligonucleotides used in this study.

Name	Sequence (5'→3')
**T7**	TAATACGACTCACTATAGGG
**S3510**	AATTCTTAAGAAGGAGATATATACATATGGACCATGATTACGCATCATCAT CATCATCATG
**S3511**	GATCCATGATGATGATGATGATGCGTAATCATGGTCATATGTATATATCTCCTTCTTAAG
**S3525**	AAAAAATCGATGTTTACCTAACGAGTGCAAAA
**S3526**	AAAAAAGAATTCCGCGCCCAGGGGCTACGGA
**S3527**	AAAAAAATCGATGCATGCTGCGCCACCATG
**S3528**	AAAAAAATCGATAAATCAACGGCCAGCGAGGTG
**S3859**	AACGCATGCAAAAACGCCCGGTGATCTCGGGGCGTTTTT
**S3860**	AAAAACGCCCCGAGATCACCGGGCGTTTTTGCATGCGTT
**S8325**	AAAAAACCGGTGATATCAGGAGAGCGTTCACCGA
**S8326**	AAAAAACTGCAGGATCATGGAACTACCCTATGC
**S8398**	Phosphate-ACACGCTGAACTTGTGGCC
**S8399**	CCATCCTGGTCGAGCTGG
**S8400**	GAACAGCTCCTCGCCCTT
**S8449**	AAAAAAAAGCTTTCAGGCCAGGTTTTGTTCCG
**S8464**	AAAAAAGGATCCACCCGAGCAACGCGAATCA
**S8471**	AAAAAACATATGACCCGAGCAACGCGAA
**S8485**	GCATGGTGGAGCAGATCG
**S8486**	TTGTCACAACCCCGTTTGAA
**S8533**	Cy5-TCCTTCTTAAGAATTCCGCG
**S8534**	TCCTTCTTAAGAATTCCGCG
**S8779**	AAAAAAGGATCCGGCCAGGTTTTGTTCCGTCA
**S8923**	AAAAAAATCGATCGAGTGCAAAAAAGTATCAGT
**S8924**	AAAAAAATCGATAAAGTATCAGTCCAAGTGCTC
**S8925**	AAAAAAATCGATTCCAAGTGCTCCCAAGGATT
**S8926**	AAAAAAATCGATCCCAAGGATTTGTCACAACC
**S8984**	AAAAAAATCGATAGTGCAAAAAAGTATCAGTCC
**S8985**	AAAAAAATCGATGCAAAAAAGTATCAGTCCAAG
**S8986**	AAAAAAATCGATCGtcacgAAAAAAGTATCAGTCCAAGTGC
**S8987**	AAAAAAATCGATCGAGTGCttttttGTATCAGTCCAAGTGCTCCC
**S8988**	AAAAAAATCGATCGAGTGCAAAAAAcatagAGTCCAAGTGCTCCCAAGG
**S8989**	AAAAAAATCGATCGAGTGCAAAAAAGTATAGTCCAAGTGCTCCCAAGG
**S9013**	AAAAAAATCGATCGAGTGCAAAAAAGTATCtcaggAAGTGCTCCCAAGGATTTGT
**S9014**	AAAAAAATCGATCGAGTGCAAAAAAGTATCAGTCCttcacCTCCCAAGGATTTGTCACAA
**S9015**	AAAAAAATCGATCGAGTGCAAAAAAGTATCAGTCCAAGTGgagggAAGGATTTGTCACAACCCCG
**S9016**	AAAAAAATCGATAAAAAAGTATCAGTCCAAGTGC
**S9017**	AAAAAAATCGATAAAAGTATCAGTCCAAGTGCT
**S9303**	AAAAAAATCGATCGAGTGCAAAAAAGTATCAGTCCAAGTGCTCCCttcctaTTGTCACAACCCCGTTTGAA
**S9377**	CGCCTCGATCCACTGGG
**S9383**	CTACGGGGAGTCAGGAAG
**S9592**	AAAAAAATCGATCGAGTGCAAAAAAGTATCAGTCCAtcacgTCCCAAGGATTTGTCACAAC
**S9593**	AAAAAAATCGATCGtcacgAAAAAAGTATCAGTCCAtcacgTCCCAAGGATTTGTCACAAC
**S9711**	Cy5-GTGGTTGCTGCTTGGGATA
**S10177**	CGAGTGCAAAAAAGTATCAGTCCAAGTGCaaaaAAGtATTTGTCACAACCCCGTTTGAA
**S10195**	AAAAAAATCGATAGTGCaaaaAAGtATTTGTCACAACCCCGT
**S10215**	AAAAAATCGATCGAGTGCAAAAAAGTATCAGTCCAAGTGCTCCCAAGGATagGTCgCAACCCCGTTTGAAGGCT
**S10216**	AAAAAATCGATCGAGTGCAAAAAAGTATCAGTCCAAGTGCTCCCAAGGATTTGTCgCAACCCCGTTTGAAGGCT
**S10217**	AAAAAATCGATCGAGTGCAAAAAAGTATCAGTCCAAGTGCTCCCAAGGATTgGTCgCAACCCCGTTTGAAGGCT
**S10272**	FITC-TCCTTCTTAAGAATTCCGCG

Restriction sites are underlined. Mutations compared to the wild type are typed in lowercase.
