# Supplementary material for: Functional Characterization of the Mannitol Promoter of Pseudomonas fluorescens DSM 50106 and Its Application for a Mannitol-Inducible Expression System for Pseudomonas putida KT2440
Source: PLoS One. 2015 Jul 24;10(7):e0133248. doi: 10.1371/journal.pone.0133248 (PMC4514859; doi:10.1371/journal.pone.0133248)
Supplement: S2 Table — (PDF) [file pone.0133248.s007.pdf]

TABLE S2. PCRs used for EMSA and DNaseI Footprinting experiments.

PCR	Template plasmid	Oligonucleotides	Fragment size (bp)
**210**	pJOE7771.1	S8485/S8533[a]	188
**211**	pJOE7771.1	S8485/S8534	188
**250**	pJH215.1	S8485/S8533[a]	178
**251**	pJH219.1	S8485/S8533[a]	176
**252**	pJH220.1	S8485/S8533[a]	173
**253**	pJH228.1	S8485/S8533[a]	171
**254**	pJH229.1	S8485/S8533[a]	169
**255**	pJH216.1	S8485/S8533[a]	168
**256**	pJH217.1	S8485/S8533[a]	158
**257**	pJH218.1	S8485/S8533[a]	148
**258**	pJH210.1	S8485/S8533[a]	137
**259**	pJH221.1	S8485/S8533[a]	178
**260**	pJH222.1	S8485/S8533[a]	178
**261**	pJH223.1	S8485/S8533[a]	178
**262**	pJH224.1	S8485/S8533[a]	178
**263**	pJH225.1	S8485/S8533[a]	178
**264**	pJH226.1	S8485/S8533[a]	178
**265**	pJH227.1	S8485/S8533[a]	178
**266**	pJH230.1	S8485/S8533[a]	178
**269**	pJH233.1	S8485/S8533[a]	178
**270**	pJH234.1	S8485/S8533[a]	178
**271**	pJH210.1	S8485/S8534	137
**272**	pJOE7771.1 (Footprint forward)	S9383/S9711[a]	240
**273**	pJOE7771.1 (Footprint reverse)	S9377/S8533[a]	228
**294**	pJH253.7 (Footprint forward)	S9383/S9711[a]	228
**295**	pJH253.7 (Footprint reverse)	S9377/S8533[a]	216
**296**	pJH255.1 (Footprint forward)	S9383/S9711[a]	206
**297**	pJH255.1 (Footprint reverse)	S9377/S8533[a]	194
**300**	pJH215.1	S8485/S10272[b]	178
**301**	pJH253.7	S8485/S10272[b]	176
**302**	pJH255.1	S8485/S10272[b]	154

[a] Cy5-labelled.

[b] FITC-labelled.
